# Management of male urinary incontinence

**DOI:** 10.4103/0970-1591.65398

**Published:** 2010

**Authors:** Katie C. Moore, Malcolm G. Lucas

**Affiliations:** Department of Urology, Royal Preston Hospital, London, UK; 1 Morriston Hospital London, UK

**Keywords:** Male incontinence, post-prostatectomy incontinence, management

## Abstract

The majority of male urinary incontinence seen is secondary to sphincter weakness following prostatic surgery. As there is a rising elderly population and increasing numbers of surgical interventions for prostate cancer, incidence of male incontinence is increasing. Hence, management of male incontinence has become a subject of increased interest for urologists. Various non-surgical and surgical approaches have been suggested for this devastating condition. Non-invasive therapies are suggested for early postoperative and mild incontinence. For surgical treatment the artificial urinary sphincter is still labeled the gold standard despite the introduction of several more minimally invasive treatments. However, as yet there is no consensus on the optimal timing and best modality for managing these men. Well designed, centrally funded clinical trials are required to establish which treatment modality to offer and when in the broad spectrum of male incontinence. This review focuses mainly on the management of post-prostatectomy incontinence since the management of other types varies little from the modalities of treatment in women.

## INTRODUCTION

Male urinary incontinence is becoming increasingly prevalent so its management has become a subject of increased interest for urologists. As in women, the underlying pathophysiology is related to either detrusor over activity or sphincter weakness, or a combination of the two. Risk factors include associated bladder outlet obstruction, neurological disease, cognitive impairment and previous prostatectomy. The rising elderly population and increasing numbers of surgical interventions for prostate cancer mean that the incidence of post prostatectomy incontinence (PPI) is rising, especially in countries where these operations are commonly performed.

The UrEpik study reported the prevalence and health status associated with male urinary incontinence in a population based, multicenter study, across four cities (Auxerre, Birmingham, Nijmegen and Seoul). In this study of 4979 questionnaire responders, up to 16.3% of men reporting mild to severe incontinence in one centre, whilst 25.9% of men visited the doctor with this problem and 5.9% of men occasionally wore pads.[1] Another large community study in Leicestershire, England, found that 8.9% of men who responded to a postal questionnaire complained of urinary incontinence and the prevalence steadily increased with age.[2] For Medicare patients the rate of visits for medical attention for male urinary incontinence increased by 77% from 1992 to 1998 and annual expenditure per person in men with urinary incontinence is more than double that in men without incontinence.[3] Similarly, in the UK there were over 3000 outpatient attendances with incontinence between 2007-2008 compared with only 1000 attendances in 2003-2004.[4]

Detrusor over activity occurs in about 75% of men with benign prostatic hyperplasia and can occur in the absence of obstruction.[5] In the majority of patients, relieving the outflow obstruction improves the detrusor over activity. 10% of patients show no improvement, possibly because the instability arose de novo and was not at all related to the obstruction or because the patient had some pre-existing occult neuropathy.

The incidence of incontinence following transurethral resection of the prostate or simple open prostatectomy varies between 1 and 5%. The reported incidence of PPI has a much wider range, 8 to 77%.[6] It is known that the incidence of PPI depends both on how incontinence is defined, timing since surgery and also on who asks the question – patient reported PPI rates to independent observers exceed those reported to surgeons. Possible risk factors identified for PPI include age, prostate volume, history of TURP, volume of urine leaked on removal of catheter and features of the surgical technique including nerve sparing and the technique of bladder neck reconstruction, though each with contradictory results in the literature.[7]

Data from the largest multicenter trials and prostate cancer databases suggest that after radical prostatectomy 8-20% of patients have persistent significant post prostatectomy incontinence.[8–10] In a review of complications in a sample of 757 Medicare patients who had undergone a radical prostatectomy, 41% of survey respondents stated that urine dripped daily, 31% needed pads, adult diapers or a penile clamp for protection and 6% required another surgical intervention for urinary incontinence.[11]

Despite these very high figures, it appears that only 6-7% of patients currently undergo subsequent surgical treatment for post prostatectomy incontinence.[12,13] The implication of this is that a significant number of men remain incontinent and essentially untreated. This review focuses mainly on the management of post-prostatectomy incontinence since the management of other types varies little from the modalities of treatment in women.

## EVALUATION OF THE INCONTINENT MALE

Management of the incontinent male patient depends on an accurate diagnosis. Most incontinent male patients have outlet incompetence secondary to urethral sphincter damage. Detrusor factors contributing to incontinence are common, though rarely the sole cause. These factors must be identified or excluded so a rational management plan can be arranged.

An accurate history should be taken, detailing the nature of the incontinence (time of onset, duration, evolution, cause, number of pads used), any previous surgical procedures, previous radiation therapy, any neurological symptoms and illnesses and medications. It is important to assess the impact of the incontinence on daily activities and the extent to which the patient is bothered by the incontinence. Associated symptoms that warrant further investigation include dysuria, hematuria, recurrent urinary tract infections and any associated voiding dysfunction.

Examination includes a full urological examination, palpating the abdomen for a full bladder (overflow incontinence) and an evaluation of mental and neurological status, in particular the S2-4 spinal segments by way of measuring anal sphincter tone, perianal sensation and the bulbo-cavernosal reflex. Cystoscopy should be performed to evaluate any sphincter damage / impairment and exclude any urethral or bladder neck strictures. Although stricture may not be the cause of incontinence it may need treatment prior to any further incontinence investigation and management. The bladder should be examined to exclude a bladder stone, tumor or diverticulum, which might be exacerbating the problem.

In a study of 215 men with PPI, only 40% had genuine stress incontinence alone and 60% had a major component of bladder dysfunction contributing to their urinary incontinence, therefore, urodynamics should always be performed.[14] The purpose of the urodynamics is to identify, as clearly as possible, the cause of the leakage and also to assess other parameters which may potentially affect the success rate of future intervention. Filling studies, with provocative maneuvers to simulate the incontinence, and a voiding study should be performed. The presence of a urethral line may mask incontinence therefore a second fill followed by removal of the catheter is often essential. Indeed, in developing a protocol for urodynamics in the male with post prostatectomy incontinence, Huckabay *et al*, found that 35% of men leaked only after removal of the filling catheter. They also found that the ALPP may be significantly higher when obtained with the urethral catheter in place.[15]

Screening fluoroscopy allows visualization of the bladder outlet during storage, stress activities and voiding. It should be noted that stress incontinence in a post prostatectomy patient is often not shown with the cough when the pelvic floor contracts, but following a cough when the pelvic musculature relaxes and small movements are enough to cause leakage through a weak sphincter. Assessment of the pressure / flow relationship is very important as again this could hint to the presence of stricture disease, which would need management prior to definitive treatment for the incontinence.

The evaluation of the incontinent male as recommended by The International Consultation on Incontinence is summarized in Table 1.[16]

**Table 1:** Evaluation of the incontinent male[16]

History
Physical examination
Urinalysis
Urine culture
Post void residual (by ultrasound)
Voiding diary (2-7 days)
Pad test
Cystourethroscopy
Multichannel urodynamics

## MANAGEMENT OF THE INCONTINENT MALE PATIENT

**Figure 1:**
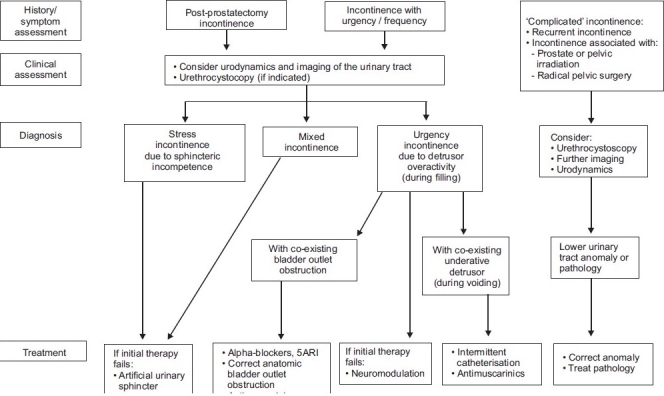
Specialised management of urinary incontinence in men

The European Association of Urologists (EAU) developed guidelines, based on the ICUD consensus from 2005, for the management of male incontinence [Figure 1]. Conservative management describes any treatment that does not involve pharmacological or surgical intervention and is considered to be simple and low cost and can be implemented at the primary care level. Many conservative management interventions require a change in behavior, which is not easy to initiate or maintain, though most patients with mild to moderate symptoms wish to try less invasive therapies first. However, patients with complicated severe symptoms may need to be referred for specialized management. In specialized management the urologist may decide to reinstate previous therapy if thought to be inadequate. If this fails then the algorithm above is used.[17]

### Non-surgical management

Non-invasive therapy should be the first line in the management of the early (i.e. the first 6-12 months) incontinence that follows a prostatectomy. Pelvic floor therapy (PFT) is the most widely recommended non-invasive treatment. However, the effectiveness of conservative therapies is dependent on patient motivation and compliance.

## PELVIC FLOOR THERAPY

In women there is strong evidence that pelvic floor therapy (PFT) is effective for stress, urge and mixed urinary incontinence. However, research findings are equivocal regarding the beneficial effects of pelvic floor therapy in men with post prostatectomy incontinence. Three studies found that PFT reduces the frequency and amount of urinary incontinence and the time to reach a continent status following a radical prostatectomy.[18–20] In contrast, three studies noted that there was no effect of PFT on these outcomes.[21–23] It should be noted that these studies vary considerably in intervention protocols, populations and outcome measures.

In a randomized clinical trial of PFT for lower urinary tract symptoms following a prostatectomy, 126 participants received brief instructions on exercising pelvic floor muscles before surgery and the offer of a biofeedback evaluation session one month after catheter removal. The intervention group (n=62) received an additional four weeks of pelvic floor training immediately after catheter removal. Over time, LUTS intensity and distress improved for participants in both groups but the degree of improvement did not differ between groups.[24]

Parekh *et al*, studied 38 patients post radical prostatectomy. Nineteen were referred to physiotherapists and completed PFT before and after surgery and 19 did not have formal PFT. Overall 66% of patients were continent at 16 weeks. A greater number in the treatment group regained continence earlier compared to the non-treatment group. However, they concluded that PFT has limited benefit in patients with severe urinary incontinence and that there is minimal long-term benefit of PFT training as continence rates at one year were similar in the two groups.[20]

## PHARMACOLOGICAL THERAPY

Duloxetine, a selective serotonin-noradrenalin reuptake inhibitor, is a recognized pharmacological therapy used in the management of stress incontinence in women, however there is no approved pharmacological agent for use in men. In a study of 20 men with stress urinary incontinence (15 post prostatectomy, five post cystoprostatectomy with orthotopic neobladder) 40mg duloxetine twice daily was administered for a mean of 9.4 weeks. Average daily use of incontinence pads decreased significantly (*P*<0.001) from 8.0 to 4.2. However, six patients complained of severe side-effects, mainly massive fatigue or insomnia and discontinued the duloxetine. This was a study with only a small group of patients followed up for only a short period of time.[25]

Alpha adrenergic antagonists can improve bladder outflow obstruction and are effective for only very minor degrees of incontinence. In early PPI, *de novo* urgency with or without detrusor over activity may play a role. In these patients anticholinergic medications can be considered. In a randomized controlled trial of tolterodine and tamsulosin in the treatment of men with lower urinary tract symptoms 24% of the 879 men recruited complained of urge urinary incontinence at baseline. They were randomized to receive either placebo, tamsulosin, tolterodine or tamsulosin plus tolterodine. Urge urinary incontinence was significantly improved by week 12 in the group receiving tolterodine plus tamsulosin and the tolterodine only group when compared to placebo but not in the tamsulosin group.[26] There were no other benefits in the anticholinergic groups when compared to placebo though an increased risk of side effects such as dry mouth was noted. There are concerns that men given anticholinergic therapy may develop urinary retention but there is no evidence from randomized controlled trials to support this.

## ELECTRICAL STIMULATION

Two studies have been identified that compared electrical stimulation (ES) and PFT with PFT alone in post prostatectomy incontinent patients. However, in these studies, the intensity of instructions and training methods differed. In the first study 58 men a median time of only 19 weeks from surgery were randomized to receive standard advice, intensive PFT with a physiotherapist or intensive PFT with a physiotherapist plus ES. Incontinence improved greatly in all three groups which may have masked any treatment effect.[21] In the second study, 139 post prostatectomy patients were randomized to receive PFT, PFT plus ES for 15 minutes twice daily or PFT plus ES and biofeedback. Treatment was started immediately after catheter removal and continued for three months. Overall continence rates improved from 21.4% on day 1 to 59.2% at three months to 85.9% at 12 months. There was no significant difference amongst the three groups. The concluding message was that €711 could be saved by omitting ES and biofeedback.[27]

The UK National Institute for Health and Clinical Excellence (NICE) recommends in its guidelines for women with urinary incontinence that further research into electrical stimulation is needed as if it proves not to be beneficial then it should not be offered as this technique is costly in staff time and outlay of equipment. (NICE) The evidence base for men is weaker so clearly similar research is required for this group too.[28]

## EXTERNAL APPLIANCES

Penile clamps, indwelling catheters, condom catheters and pads are occasionally used but are not generally considered socially acceptable and can be a source of anxiety and discomfort if incorrectly fitted. They should be reserved for minor degrees of incontinence or in patients who have multiple other co-morbidities in whom surgery may be thought inappropriate.

The safety, efficacy, comfort and patient satisfaction with three types of penile compression devices (C3, U-Tex Male Adjustable Tension Band and Cunningham clamp) was evaluated in a small (12 patients) randomized control cross-over trial. The Cunningham device was the most efficacious and acceptable to users, but, significantly reduced the distal blood flow velocity. None of the devices completely eliminated urine loss when applied at a comfortable pressure. Potential complications of penile clamps include edema, pain, urethral erosion and obstruction and they should not be used for more than four hours at a time.[29]

Absorbent products for male incontinence (pouches, leafs, absorbant pants, small pads) were evaluated in a multi-center, multi-crossover study. Absorbancy without leakage was the characteristic most important to users. The group concluded that no one product suits everybody though small pads came close. Washable absorbant pants for men with light incontinence may have economic advantages over other choices.[30]

Urinary catheters, either indwelling or intermittent use, can be used as a short term measure while a man awaits surgical management of his incontinence or as a permanent solution if operative management is not suitable and the incontinence cannot be managed with absorbent devices. Recurrent urinary tract infections, urethral trauma and calculi are significant complications associated with indwelling catheters. Sheath catheters were found to be significantly more comfortable, less painful, less restrictive, and more convenient causing less embarrassment than indwelling catheters, when evaluated in 104 men using a catheter in an American hospital.[31]

NICE guidelines recommend that a choice of containment products to manage urinary incontinence in women should be offered based on individual circumstances and patient preference. Devices can be offered as a means of achieving social continence prior to a definitive diagnosis and whilst a management plan is developed. Permanent continence product use should be used only after exclusion of other methods of continence management. Product preference depends on lifestyle and severity of the incontinence. Different types of product for night time versus day time use and when going out versus staying in may be preferred. Similar guidance can be applied to incontinent men.[28]

### Surgical therapies

A proportion of patients fail to respond to the above mentioned conservative therapies and in these patients a surgical approach should be considered.

## URETHRAL BULKING AGENTS

Substances injected transurethrally or periurethrally to augment the urethral wall and increase urethral resistance to urinary flow include Teflon paste, autologous fat, collagen and silicon macroparticles. Enthusiasm for urethral bulking agents has fallen because of the low long term success rates associated with the procedure and the need for multiple injections. Prior to any injection therapy, concurrent detrusor over activity should be managed. A study by Martins *et al*, of 46 patients with postprostatectomy incontinence injected with collagen had an overall improvement rate of 65%. Of those that failed to improve, 79% had detrusor instability.[32]

Bovine gluteraldehyde cross linked (GAX) collagen was introduced in 1993 and has been widely used as a urethral bulking agent in the management of intrinsic sphincter deficiency in men. It is a safe technique which can be repeated several times and induces only a minimal inflammatory response and has not been shown to migrate unlike the Teflon paste.[33] Abosief *et al*, evaluated 88 patients who had a mean of 3.5 injections and 25ml of collagen. At 10 months, 48% were dry or nearly completely dry and a further 38% improved. Patients required up to five injections with some not finding an improvement until after the forth injection.[34]

In a study from Texas 322 men with intrinsic sphincter deficiency received transurethral collagen injections. Overall the mean number of injections was 4.37. Mean duration of response was 11.1 months in those who achieved complete continence (17%). It was concluded that transurethral collagen injections are a good non-invasive option but only in the short term for men with post prostatectomy incontinence.[35] Use of this product is now declining because of the low success rates and the need for multiple treatments.

A study from Finland evaluated the effects of Macroplastique injection on post operative stress urinary incontinence in a series of 50 consecutive men. They were suffering from mild to moderate SUI and had 2.5 to 5ml of Macroplastique injected adjacent to the external sphincter at 5 or 7 ‘o’ clock or both. At baseline mean, one-hour-pad test-loss was 48.3ml. After the first injection six patients were completely dry, 28 improved and the remainder had no effect. Forty patients underwent a second injection after which 10 more became completely dry and five improved significantly. Twenty three patients had a third injection and 8 required a forth injection.[36]

## ARTIFICIAL URINARY SPHINCTER

The artificial urinary sphincter (AUS) is considered the gold standard treatment for stress urinary incontinence after a prostatectomy, offering the patient the greatest chance of a cure.[16,17] The AUS was developed by Scott *et al*, and first introduced in 1973. Its basic design has undergone several changes and improvements to become the current device, the AMS 800.[37] This consists of an occlusive cuff to be placed around the bladder neck or bulbar urethra, a pump mechanism, placed in a dependant superficial position in the scrotum which can be easily activated and deactivated and a reservoir all connected by kink resistant tubing. The sphincter mechanism is filled with isotonic contrast medium to prevent fluid loss through osmotic transfer across the silicone membrane of the reservoir and to allow radiological evaluation of any leaks.

Prior to insertion of the artificial sphincter, urodynamic studies should be performed as a proportion of patients will have abnormal bladder storage which should be treated appropriately prior to insertion of the sphincter. Cystoscopy should be performed to exclude any stricture disease. It is essential that there is no obstruction prior to AUS insertion as subsequent treatment can become difficult or even impossible meaning strictures need to be treated and stable prior to insertion of an AUS. The patient needs to have the dexterity and mental faculties to manage the sphincter and may require careful counseling regarding the long term management of the sphincter.

There is a large amount of data on continence rates, complications and patient satisfaction following implantation of the AMS 800. The largest report to date examines 323 patients who had the AMS 800 implanted at the Mayo clinic. At a mean follow up of 68.8 months, continence rates of 79% were achieved. 27% of the men, however, required reoperation for mechanical failure of the device.[38]

Satisfaction rates following implantation of an artificial urinary sphincter are high. In a study by Litwiller *et al*, 90% of patients reported satisfaction with the AUS and 96% stated they would recommend the artificial urinary sphincter to a friend. In retrospect 92% of the patients would have an AUS placed again and 90% of those undergoing revision reported no change in satisfaction.[39]

Problems relating to the implantation of an artificial sphincter include patients’ inability to manage the mechanism correctly, urethral atrophy, loss of fluid from the mechanism, cuff erosion and mechanical failure of the device. The most feared complication is cuff erosion (1-3%) necessitating complete removal of the system.[38] Erosion can be precipitated by infection, excessive cuff pressure, previous radiotherapy, too small a cuff resulting in decreased vascularity and trauma via catheterization through an activated cuff. AUS infection rates are 2-10%. The revision rate is 9% with 15 year survival of the AUS expected to be 75%.[40] Recurrent urinary incontinence after AUS insertion is commonly due to urethral atrophy. This occurs in 3-9% of patients and is managed by either down sizing or repositioning the cuff.[39]

Intrinsic difficulties with the AUS include the problem that it is not a stress responsive system – the closure pressure is constant and this means that momentary increases in abdominal pressure will still result in leakage if they exceed that closure pressure. It is not possible to achieve a change in resting closure pressure without reoperation to increase reservoir pressure or implant a different size cuff. The morbidity and risk of failure of repeat operations tends, unsurprisingly, to increase progressively. The “Flowsecure” is an artificial urinary sphincter with conditional occlusion, designed to provide good continence rates adjusting regulating pressures when needed and conceived to reduce the risk of potential complications associated with excessive occluding pressures and mechanical failure. A stress relief balloon transmits transient increases in abdominal pressure to the cuff during periods of stress.[41]

The use of a tandem cuff has been reported in men with severe stress urinary incontinence. This offers the advantage of pressure exertion of a greater urethral length thereby increasing resistance to urinary leakage. O’Connor *et al*, evaluated outcomes in a cohort of men who received a double cuff implant compared with a matched cohort with a single cuff AUS. Initially, at 2 years follow up, the double cuff AUS provided improved dry rates with complication rates comparable to the single cuff. At an average of 71 and 58 months follow-up for the single and double cuffs respectively, there was no statistically significant difference in continence rates between the two groups. Furthermore, men receiving double cuff implants were thought to be at a higher risk of complications requiring further surgery.[42]

## URETHRAL SLINGS

The first use of urethral slings in males was the use of rectus fascial slings in post prostatectomy patients.[43] The advantage of the male sling compared to the AUS is that in theory it allows spontaneous voiding without the need for manipulation and it provides instantaneous results to the patient (there is a need for deactivation of the AUS device for four to six weeks after implantation). Also, slings can be used in patients that have limited manual dexterity and an AUS can still feasibly be inserted at a later date if required. The male sling compresses only the ventral aspect of the urethra leaving the dorsal and lateral blood flow intact (compared to the AUS which compresses the urethra circumferentially predisposing to atrophy and erosion). Infection and erosion rates for the perineal sling are low (in a study of 49 consecutive patients over four years three patients developed infection and there were no cases of erosion).[44]

The bone anchored sling uses six, 5mm titanium screws that are drilled into the anteromedial aspects of each descending pubic ramus using the InVance bone drill (American Medical Systems). These screws are preloaded with a pair of number one polypropylene sutures. A propylene mesh alone or in combination with dermis as a composite graft is used as a sling material. After one side of the sling is anchored to the pubic ramus, sling tension is adjusted either by retrograde leak point pressure measurement or asking the patient to cough if they are awake. The sling is then tied down on the opposite pubic ramus. Comiter presented the results of 48 patients who all rated their incontinence as severe and used >3 pads per day prior to bone anchored sling surgery. Median follow-up was 48 months with mean pad usage decreasing from 4.6 pads per day to 1.0 pad per day. Overall 31/48 (65%) patients were cured of their incontinence, 7/48 were very much improved 3/48 were mildly improved and 7/48 failed. Complications included one infection leading to erosion, 7 cases (16%) of bothersome scrotal pain / numbness all of which had resolved by three months and two cases of bone screw dislodgement.[45]

The Remeex Male Readjustable System^®^ (MRS, Neomedic International, Spain) is composed of a monofilament sub urethral sling connected via two monofilament traction threads to a suprapubic mechanical regulator. The mechanical regulator is a permanent subcutaneous implant which allows adjustment of sub urethral pressure from outside the body by means of an external manipulator. In a multicenter European study with a mean follow-up of 32 months, 33/51 (64.7%) of patients were cured following implantation of this type of sling. Almost all patients required at least one adjustment of the sling performed under local anesthetic. Three patients needed the sling removed – one for urethral erosion and 2 for infection of the regulator. Intra-operative bladder perforation occurred in five patients. Perineal discomfort or pain was very common and managed with simple analgesia.[46]

The male polypropylene retrourethral sling (AdVance, American Medical Systems) passes through the obturator foramen on both sides, underneath the adductor longus tendon and around the proximal urethral bulb. Tensioning the sling by pulling on it on both sides as they exit the obturator foramina exerts a rotational movement of the posterior surface of the bulb in a proximal direction. This technique has been promoted as having a fundamentally different mode of action to other types of sling in that it is non-obstructive, repositioning the sphincter to its preoperative position, but it remains unclear as to whether this is a real difference or merely an attractive hypothesis with considerable marketing benefits. Following a cadaveric study in which implantation of this device was found to achieve a leak point pressure of 60 cm H_2_O, Rehder and Gozzi implanted the device in 20 men with PPI. Incontinence cure rate was 40% and a further 30% improved. Q_max_ was not changed by insertion of the sling and 12/20 men were satisfied with the outcome.[47]

Kumar *et al*, found that when given the option of either an AUS or a sling, the opportunity to avoid a mechanical device was preferable to a well established treatment. All patients who were recommended a sling procedure took it. When an AUS was recommended 75% chose it and 25% opted for the sling. When given a choice 92% of men chose the sling and 8% chose the AUS. Men who were strongly willing to avoid a mechanical device were prepared to go against the surgeon recommendation for an AUS.[48]

## PROACT

The ProAct device (Uromedica Inc) is a minimally invasive treatment for male stress urinary incontinence. It is composed of two silicone elastomer balloons placed paraurethrally at the bladder neck in post radical prostatectomy patients or at the level of the membranous urethra in me who have residual prostatic surgery. Each balloon is attached to a titanium port which is buried in the anterolateral aspect of the scrotum. If required, these ports allow adjustment of the balloon pressures post-operatively to achieve the desired urethral resistance without further surgical intervention. A further advantage of the ProAct device is that it can be easily removed if the balloons prove to be painful, erosive or become infected.[49]

About 117 patients were implanted with the ProAct device between 1999 and 2004 in one center. Implantation took 14-56 minutes with the last 20 all being completed within 25 minutes as experience increased. On the first day post insertion only five men were fully continent and needed no further percutaneous adjustment of the balloon volume. 112 (96%) needed a median (range) of three (1-15) adjustments to achieve a satisfactory result. Median balloon volume at implantation was 2ml (0.5-7.5ml) and the final mean volume after adjustments was 3.5ml (1-10ml). In 15 men there were perforations, (bladder / urethral), at the time of surgery. At one year 92% of men were wearing fewer pads compared to baseline and 88% were described as continent or mildly incontinent. From this series 28 have now had an AMS 800 AUS implanted. Five of these had opted for a male sling but this also proved insufficient to manage their incontinence leading them to accept an AUS.[49]

Crivellaro *et al*, compared the efficacy of the adjustable continence therapy (ProAct) with the bone anchored male sling (BAMS) in men with post prostatectomy incontinence; 46 men received the ProAct and 38 had the BAMS implanted by two different operators in two different centers. Both groups were followed up prospectively by number of pads used per day and the UCLA/RAND questionnaire. Complication rates and operating times were also compared. At 19 months post insertion, 30/44 (68%) of the ProAct patients were dry and at 33 months 23/36 (64%) of the BAMS patients were dry. The UCLA / RAND questionnaire showed an average increase of 11.7 points for ProAct and of 10 points for BAMS. Complications included removal of the ProAct or BAMS in 6/44 (14%) and 2/36 (6%) respectively. Mean operation time was 18 minutes for the ProAct and 45 minutes for the BAMS. They concluded that the ProAct and BAMS are both associated with a satisfactory outcome and that the ProAct results seemed to be better for more severe incontinence and BAMS for mild incontinence.[50]

## BLADDER NECK / URETHRAL CLOSURE

Closure of the bladder outlet with placement of a suprapubic catheter or, if appropriate, construction of a continent catheterizable stoma for bladder emptying is an alternative to cystectomy with urinary diversion. The traditional method of bladder outlet closure in men is either by way of the perineum, closing the urethra below the prostatic apex, or suprapubically closing the urethra at the bladder neck.[51] Only a few studies have specifically examined the outcomes in men after bladder neck closure. Shpall reported the long-term results of 21 men who underwent suprapubic bladder neck closure. Four patients (19%) developed a bladder neck fistula.[52] In a cohort of 23 males who underwent suprapubic bladder neck closure over a period of 10 years, 4 had continued leakage from the bladder neck visualized on cystoscopy. Two of these underwent successful closure with a second transabdominal procedure. Two failed at a second attempt at bladder closure and underwent a simple cystectomy and conduit diversion. Complications included fistula formation, stomal stenosis and formation of bladder calculi. O’Connor attempted perineal bladder neck closure in two men, but this treatment failed in both.[51]

This is a procedure largely reserved for patients with multiple sclerosis in whom long term management with a urethral catheter has resulted in continued urethral leakage resulting usually from overactive bladder contractions.

## CONCLUSION

The ICUD states that multichannel urodynamics are essential prior to any invasive treatment for urinary incontinence.[16] After a period of conservative management lasting 6 to 12 months operative intervention is to be considered. However, there are still no strong recommendations for the ideal management and no conclusive data regarding the optimal time to begin treatment.

If non-invasive therapy fails, surgical options are recommended. For severe or persistent incontinence, the insertion of an AUS is still the gold standard as it appears to be associated with high patient satisfaction and high continence rates. Several more minimally invasive treatments have been introduced in recent years with varying degrees of success but they are yet to surpass the results of the AUS. Nevertheless, patient demand for minimally invasive treatment options is high and poorer continence results may be accepted by the patient to avoid an AUS.

Well designed, centrally funded (as opposed to those funded by manufacturers) clinical trials are required to determine which treatment modality to offer in the broad spectrum of male incontinence. Issues particularly relating to adverse events may be best addressed by the advent of central registers such as those offered by the British Association of Urological Surgeons. It is clear that there is a need for centralization of PPI surgery into units that are able to offer a complete range of surgical options and have a sufficient workload to develop and maintain expertise and to participate in the studies that will establish the relative clinical utility of each procedure.
